# Syntheses, Characterization, Resolution, and Biological Studies of Coordination Compounds of Aspartic Acid and Glycine

**DOI:** 10.1155/2017/2956145

**Published:** 2017-02-15

**Authors:** Temitayo Aiyelabola, Ezekiel Akinkunmi, Isaac Ojo, Efere Obuotor, Clement Adebajo, David Isabirye

**Affiliations:** ^1^Department of Chemistry, Obafemi Awolowo University, Ile-Ife, Osun State, Nigeria; ^2^Department of Pharmaceutics, Obafemi Awolowo University, Ile-Ife, Osun State, Nigeria; ^3^Department of Biochemistry and Molecular Biology, Obafemi Awolowo University, Ile-Ife, Osun State, Nigeria; ^4^Department of Pharmacognosy, Obafemi Awolowo University, Ile-Ife, Osun State, Nigeria; ^5^Department of Chemistry, North-West University, Mafikeng Campus, Mmabatho, South Africa

## Abstract

Enantiomerically enriched coordination compounds of aspartic acid and racemic mixtures of coordination compounds of glycine metal-ligand ratio 1 : 3 were synthesized and characterized using infrared and UV-Vis spectrophotometric techniques and magnetic susceptibility measurements. Five of the complexes were resolved using (+)-*cis*-dichlorobis(ethylenediamine)cobalt(III) chloride, (+)-bis(glycinato)(1,10-phenanthroline)cobalt(III) chloride, and (+)-tris(1,10-phenanthroline)nickel(II) chloride as resolving agents. The antimicrobial and cytotoxic activities of these complexes were then determined. The results obtained indicated that aspartic acid and glycine coordinated in a bidentate fashion. The enantiomeric purity of the compounds was in the range of 22.10–32.10%, with (+)-*cis*-dichlorobis(ethylenediamine)cobalt(III) complex as the more efficient resolving agent. The resolved complexes exhibited better activity in some cases compared to the parent complexes for both biological activities. It was therefore inferred that although the increase in the lipophilicity of the complexes may assist in the permeability of the complexes through the cell membrane of the pathogens, the enantiomeric purity of the complexes is also of importance in their activity as antimicrobial and cytotoxic agents.

## 1. Introduction

The need for more potent antimicrobial and anticancer agents has led to increased attention given to coordination compounds in recent times. This is partly due to the reports of increased bacterio- and carcinostatic activities of some biologically active compounds upon chelation [1–5]. A current challenge facing coordination chemists is that of obtaining enantiomerically pure compounds [6–11]. Since the potential target receptor sites for pharmacological agents are biopolymers with chiral subunits, the activity of therapeutic agents may involve molecular recognition by these sites, thereby demanding high optical purity [12–17]. Hence, high enantiomeric purity is essential for the effectiveness of these agents and it is therefore important that these agents are enantiopure. Research has shown that when an enantiomer of a racemic or enantiomerically enriched form is inactive, the potency of the active enantiomer is reduced, similar to the concept of deliberate adulteration. Such situations were encountered with warfarin and ibuprofen [12, 15–19]. In some cases, one of the enantiomers may have toxic side effects, as typified by thalidomide. Both enantiomers of thalidomide have desirable sedative properties; however, the (−)-enantiomer is teratogenic, presenting toxic side effects that led to its withdrawal from the market [20, 21]. Therefore, all these problems dictate the need of these optically active compounds as enantiopure compounds. Resolution serves as a way of discriminating between enantiomers of coordination compounds and diastereoisomer formation is the primary technique used [22–30]. There is no single resolving agent that can be considered universal. Hence, choosing the right resolving agent for enantiomeric separation may be a herculean task [13, 27].

**Figure 1:**
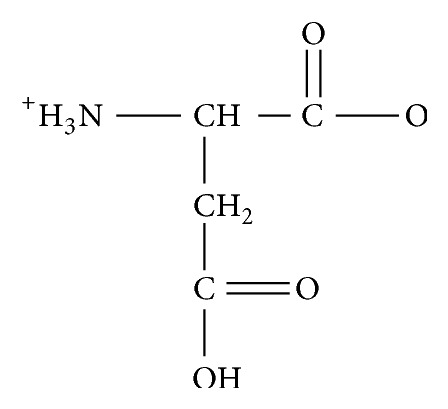
Aspartic acid.

**Figure 2:**
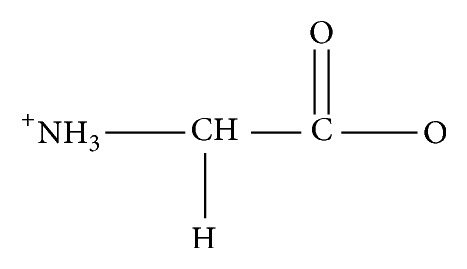
Glycine.

Previous workers have synthesized and reported some significant antimicrobial and cytotoxic activities of coordination compounds of various amino acids [31–37]. However, these studies on their enantiomerically enriched and pure forms are few [38] and, as indicated above, obtaining these compounds at high enantiomeric purity may enhance their potency and reduce their toxicity. Aspartic acid (Figure 1) is one of such amino acids studied; it possesses three potential donor sites (one amine and two carboxyl groups) [37, 39, 40]. It is therefore capable of coordinating as a bi-, tri-, and monodentate and bridging ligand. Thus, a variety of geometries are possible with this ligand, which may be studied by comparing the complexes it forms with a series of metal ions of the same valency at relevant pH ranges [41–47]. Hence, in this work, coordination compounds of aspartic acid (L_1_) and glycine (L_2_, Figure 2) were synthesized and characterized. The aspartato complexes were synthesized using asymmetric synthesis via chiral pool syntheses while ion exchange chromatography was used in separating some of the compounds into their geometric isomers. In determining the most active resolving agent, the predominant isomers were further resolved using (+)-*cis*-dichlorobis(ethylenediamine)cobalt(III) chloride, (+)-bis(glycinato)(1,10-phenanthroline)cobalt(III) chloride, and (+)-tris(1,10-phenanthroline)nickel(II) chloride. The synthesized compounds and resolved complexes were then screened for their in vitro antimicrobial activity and brine shrimp lethality.

## 2. Materials and Methods

### 2.1. General

All reagents and solvents used were of analytical grade. Melting points or temperatures of decomposition were measured using open capillary tubes on a Gallenkamp (variable heater) melting point apparatus. The UV-Vis spectra were obtained using a Genesis 10 UV-Vis spectrophotometer at the Central Science Laboratory, Obafemi Awolowo University, Ile-Ife, Nigeria. The infrared spectra (KBr) were recorded on a Genesis II FT-IR spectrophotometer at North-West University, Mafikeng Campus, South Africa. Optical rotations were measured using Atago Polax-2L polarimeter at the Department of Pharmaceutical Chemistry, Obafemi Awolowo University, Ile-Ife. Magnetic susceptibility was obtained using a Sherwood scientific balance, Kwara State University, Nigeria. Antimicrobial activities of the complexes was determined at the Department of Pharmaceutics, Obafemi Awolowo University, Ile-Ife. Brine shrimp lethality bioassay was carried out at the Department Biochemistry and Molecular Biology, Obafemi Awolowo University, Ile-Ife.

The syntheses, characterization, and the antimicrobial studies for the glycinato complexes have been reported previously [33]. However, the cytotoxic activity is reported here for the first time.

### 2.2. Syntheses of Complexes

#### 2.2.1. Preparation Sodium Tris(aspartato)cuprate(II), Na[Cu(L_1_)_3_]

Copper(II) sulphate anhydrous solution (3.12 g, 0.02 M) was dissolved in 10 mL of distilled water and warmed until a clear solution was obtained. (+)–Aspartic acid solution (8.11 g, 0.06 M) was dissolved in distilled water (25 mL) and warmed over a steam bath and Na_2_SO_4_ solution (0.71 g, 0.005 M) was added with stirring. The copper sulphate solution was then added and the mixture heated for 2 hours on a steam bath (pH of the reaction was 2.31). The solution obtained was concentrated and allowed to cool overnight with the formation of a precipitate. The precipitate obtained was filtered, washed with methanol, and dried in an oven at 60°C. Yield: 6.62 g, 66.0%; d.p.: 232°C. IR data (cm^−1^): 3380, 2922, 2851, 1666, 1547, 702, 595. UV-Vis data (nm): 217, 223, 238, 262, 499, 517. Magnetic moment, *μ*_eff_(BM): 1.93.

Similar methods of preparation were used for the following compounds

#### 2.2.2. Sodium Tris(aspartato)manganese(II), Na[Mn(L_1_)_3_]

Manganese(II) chloride solution (4.38 g, 0.02 M) was added to (+)–aspartic acid solution (8.02 g, 0.06 M) and sodium sulphate solution (0.71 g, 0.005 M) gave sodium tris-aspartatomanganese(II) as white precipitate. pH of reaction 2.42; yield: 8.34 g; 83.0%; d.p.: 287°C. IR data (cm^−1^): 2920, 1681, 1506, 779, 549. UV-Vis data (nm): 217, 259, 283, 298, 550, 544, 574, 679. Magnetic moment, *μ*_eff_(BM): 5.30.

#### 2.2.3. Sodium Tris(aspartato)nickelate(II), Na[Ni(L_1_)_3_]

Nickel(II) chloride hexahydrate solution (2.32 g, 0.01 M) was added to (+)–aspartic acid solution (4.00 g, 0.03 M) and sodium chloride solution (0.58 g, 0.01 M) gave sodium tris-aspartatonickelate(II) as fine green crystals. pH of reaction 2.37; yield: 3.81 g; 88.07%; d.p.: 265°C. IR data (cm^−1^): 3497, 2994, 1527, 751, 595. UV-Vis data (nm): 241, 310, 325, 343, 541, 820. Magnetic moment, *μ*_eff_(BM): 2.83.

#### 2.2.4. Sodium Tris(aspartato)cobaltate(II), Na[Co(L_1_)_3_]

Cobalt(II) chloride hexahydrate solution (2.14 g, 0.01 M) was added to (+)–aspartic acid solution (4.01 g, 0.03 M) and sodium chloride solution (0.58 g, 0.01 M) gave sodium tris-aspartatocobaltate(II) as purple crystals. pH of reaction 2.35; yield: 3.43 g; 68.64%; d.p.: 265–267°C. IR data (cm^−1^): 3404, 2853, 1654, 1547, 724, 598. UV-Vis data (nm): 223, 241, 247, 307, 484, 505. Magnetic moment, *μ*_eff_(BM): 5.04.

#### 2.2.5. Sodium Tris(aspartato)cadmium(II), Na[Cd(L_1_)_3_]

Cadmium(II) chloride monohydrate solution (3.12 g, 0.02 M) added to (+)–aspartic acid solution (8.11 g, 0.06 M) and (0.58 g, 0.01 M) sodium chloride solution, gave sodium tris-aspartatocadmium(II) as a white precipitate; pH of reaction 2.44, yield: 6.21 g; 62.60%; d.p.: 220–222°C. IR data (cm^−1^): 3547, 3453, 2890, 1684, 1460, 776, 599. UV-Vis data (nm): 217, 238, 280, 295. Magnetic moment, *μ*_eff_(BM): 0.00.

#### 2.2.6. Sodium Tris(glycinato)cobaltate(II), Na[Co(L_2_)_3_]

Cobalt(II) chloride hexahydrate solution (2.51 g, 0.01 M) was added to glycine solution (2.29 g, 0.03 M) and sodium chloride solution (0.58 g, 0.01 M) gave sodium tris-glycinatocobaltate(II) as pink crystals. pH of reaction 2.45; yield: 1.84 g; 61.6%; d.p.: 223°C. IR data (cm^−1^): 3428, 2834, 1621, 1454, 672, 509. UV-Vis data (nm): 220, 226, 256, 520, 667, 682; Magnetic moment, *μ*_eff_(BM): 5.20.

#### 2.2.7. Bis(glycinato)copper(II) Complex, [Cu(L_2_)]_2_

Copper(II) chloride anhydrous solution (1.62 g, 0.01 M) added to glycine (2.29 g, 0.03 M) and sodium sulphate solution (0.71 g, 0.005 M) gave* bis*(glycinato)copper(II) complex as blue precipitate. pH of reaction 2.59; yield: 2.01 g; 67.00%; d.p.: 198°C. IR data (cm^−1^): 3333, 2913, 1632, 1416, 695, 501. UV-Vis data (nm): 262, 620, 632. Magnetic moment, *μ*_eff_(BM): 1.53.

### 2.3. Resolution of the Geometrical Isomers

Complexes of L_1_ and L_2_, namely, Na[Co(L_1_)_3_], Na[Cu(L_1_)_3_], Na[Ni(L_1_)_3_], [Cu(L_2_)]_2_, and Na[Co(L_2_)_3_], were separated into their geometric isomers using an adaptation of the method described by Glodjović et al. (2005) [48].

#### 2.3.1. Sodium Tris(aspartato)cobaltate(II), Na[Co(L_1_)_3_]

Sodium tris(aspartato)cobaltate(II) (2.83 g, 0.005 M) was loaded onto a column containing Dowex X 50 (200–400 mesh) anion exchange resin (chloride ion form). The column was washed with water and eluted with 0.1 M NaClO_4_. Two bands were collected and their eluates concentrated and left to crystallize. The crystals were thereafter washed and filtered. First eluted isomer (pink): yield: 1.08 g, 38.16%; d.p.: 261–263°C; UV-Vis (nm): 220, 238, 245, 332, 340, 344, 510. Second eluted isomer (blue): yield: 0.52 g, 18.37%; d.p.: 265°C; UV-Vis (nm): 226, 232, 246, 336, 340, 346, 514.

A similar method was used for the following compounds.

#### 2.3.2. Sodium Tris(aspartato)cuprate(II), Na[Cu(L_1_)_3_]

Sodium tris(aspartato)cuprate(II) (2.42 g, 0.005 M) gave only one band (blue). Yield: 1.82 g, 75.21%; d.p.: 230°C; UV-Vis (nm): 222, 232, 249, 250, 254, 340, 356, 974.

#### 2.3.3. Sodium Tris(aspartato)nickelate(II), Na[Ni(L_1_)_3_]

In sodium tris(aspartato)nickelate(II) (2.45 g, 0.005 M), one band was observed (green). Yield: 1.66 g, 67.82%; d.p.: 225°C; UV-Vis (nm): 444, 648, 980.

#### 2.3.4. Sodium Tris(glycinato)cobaltate(II), Na[Co(L_2_)_3_]

In sodium tris(glycinato)cobaltate(II) (3.05 g, 0.01 M), two bands were obtained. First eluted isomer (purple): yield: 1.87 g, 61.31%; d.p.: 261–263°C; UV-Vis (nm): 360, 444, 567. Second eluted isomer (light pink): yield: 0.21 g, 6.89%; d.p.: 265°C; UV-Vis (nm): 290, 386, 665.

#### 2.3.5. Bis(glycinato)copper(II), [Cu(L_2_)_2_]_2_

Bis(glycinato)copper(II) [Cu(L_2_)_2_]_2_ (3.11 g, 0.01 M) column eluted with water gave blue eluant. Yield: 1.92 g, 63%; d.p.: 212°C; UV-Vis (nm): 296, 386, 628. When eluted with 0.1 M, NaClO_4_ gave a violet eluant. Yield: 0.12 g, 7%; d.p.: 221°C; UV-Vis (nm): 232, 290, 386, 637,980.

### 2.4. Preparation of Resolving Agents

#### 2.4.1. *cis-*Dichlorobis(ethylenediamine)cobalt(III) Chloride

The synthesis of* cis-*dichlorobis(ethylenediamine)cobalt(III) chloride was carried out using an adaptation of the method described by Moriguchi (2000) [49]. Cobalt(II) chloride-6-hydrate (24 g, 0.1 M) was dissolved completely in distilled water (35 mL). Anhydrous ethylenediamine (15 g, 0.2 M) in 50 mL of water was then added. Hydrogen peroxide (12 mL) was added dropwise with stirring. The oxidized solution was cooled in ice and 12 mL of concentrated HCl was added with stirring. A dark ruby red solution was formed. The solution was then concentrated in a water bath and cooled. Dark green crystals were obtained. The crystals were thereafter dissolved in methanol solution, filtered, and dried in an oven at 100°C. Yield: 23.26 g; 82.23%. The green* trans*-dichlorobis(ethylenediamine)cobalt(III) chloride obtained was then dissolved in a minimum amount of water, heated, and evaporated to dryness to give a glassy deep violet product. This was filtered and washed with iced cold water to obtain a violet powder of* cis-*dichlorobis(ethylenediamine)cobalt(III) chloride. Yield: 21.31 g; 75.34%. IR (cm^−1^): 3444, 3230, 3104, 2903, 2012, 1612, 1099, 938, 801. UV-Vis (nm): 222, 245, 342, 489, 609.

#### 2.4.2. Bis(glycinato)(1,10-phenanthroline)cobalt(III) Chloride

A solution of 1,10-phenanthroline (4.4 g, 0.02 M) and glycine (3.0 g, 0.04 M) in water (50 mL) was added to cobalt(II) chloride-6-hydrate (5.83 g. 0.02 M) solution with stirring. Hydrogen peroxide was added gradually to the dark orange solution with vigorous stirring. The suspension obtained was concentrated by heating at 65°C and cooled to obtain crystals which were filtered and dried. Yield: 8.23 g, 89.20%.

The product obtained, 4.60 g, 0.01 M, was poured onto a cation-exchange resin column (Dowex 50W-X8, 200–400 mesh, anion exchange resin, chloride ion form). The orange solution of [Co(gly)_2_phe]Cl was obtained by eluting with 0.20 M KBr solution. It was then concentrated at 70°C, cooled, and filtered. The residue was dissolved in methanol and the solution was filtered. The crude complex was obtained by the addition of acetone to the filtrate. Recrystallization was achieved by dissolving it in a minimum amount of water to which methanol-acetone (1 : 1) mixture was added. This solution was allowed to stand in a refrigerator overnight. The orange crystals deposited were filtered and washed with methanol-acetone (1 : 2) mixture and dried in a vacuum oven. Yield: 2.86 g, 62.63%. IR (cm^−1^): 3001, 2858, 2721, 2650, 1870, 1685, 1596, 1507, 1417, 1304, 1245, 1149, 983, 893. UV-Vis (nm): 224, 238, 244, 507, 618.

#### 2.4.3. Tris(1,10-phenanthroline)nickel(II) Chloride

A solution of 1,10-phenanthroline (6.50 g, 0.03 M) was added to a solution of nickel(II) chloride (2.45 g, 0.01 M) with stirring. The resulting solution was heated over a water bath until a scum was observed. The product was then left to crystallize, and the crystals obtained were filtered, washed with methanol, and dried. Yield: 4.49 g, 65.24%. IR (cm^−1^): 3397, 3230, 3104, 2012, 1612, 1081, 944, 807. UV-Vis: 222, 232, 244, 342, 521, 623.

### 2.5. Resolution of Resolving Agents

#### 2.5.1. Resolution of* cis*-Dichlorobis(ethylenediamine)cobalt(III) Chloride

Potassium antimonyl-D-tartrate hydrate (6.74 g, 0.01 M) was dissolved in 10 mL of water with warming and* cis*-[Coen_2_Cl_2_]Cl (5.96 g, 0.02 M) dissolved in 20 mL of water was added. The solution obtained was heated to 80°C with stirring for 45 min. A pale violet precipitate was formed. The product was isolated by filtration, washed with ethanol and diethyl ether, and dried in a vacuum oven at 60°C. Yield: 7.11 g; 78.61%. The diastereoisomer (5.21 g, 0.005 M) obtained was added with stirring to an aqueous slurry of potassium chloride (0.52 g, 0.005 M). Methanol was then added to precipitate the* cis*-dichlorobis(ethylenediamine)cobalt(III) chloride. The residue obtained was recrystallized with methanol and acetone. Optical rotation was constant after two recrystallizations. Yield: 0.93 g; 65.24%; [*α*]^589^ = +87°. The general equations for the reactions are shown in

(1) 4CoCl2·6H2O+8en+H2O2⟶4Coen2OHH2OCl2

(2) trans-Coen2OHH2OCl2+2HCl⟶Coen2Cl2Cl·HCl+2H2O

(3) trans-Coen2Cl2Cl⟶cis-Coen2Cl2Cl

(4) 2cis-Coen2Cl2Cl+K2Sb2d-tart2⟶+-Coen2Cl22Sb2d-tart2+2KCl

(5) +-Coen2Cl22Sb2d-tart2⟶2+-Coen2Cl2Cl+K2Sb2d-tart2grind  with  KCl

#### 2.5.2. Resolution of Bis(glycinato)(1,10-phenanthroline)cobalt(III) Chloride

Potassium antimonyl-D-tartrate hydrate (6.41 g, 0.01 M) was dissolved in 10 mL of water with warming. A solution of the racemic mixture of bis(glycinato)(1,10-phenanthroline)cobalt(III) chloride (9.02 g, 0.02 M) dissolved in 20 mL of water was then added and stirred. The diastereomer which was deposited was filtered and washed with a methanol-acetone mixture (1 : 1) and then acetone. The diastereomer was recrystallized by dissolving in a minimum quantity of water, followed by gradual addition of methanol to produce turbidity, and then cooled in ice to obtain crystals which were filtered, washed, and dried.

A solution of the diastereomer (6.81 g, 0.005 M) in a minimum amount of water was added to potassium chloride (0.52 g, 0.005 M) with vigorous stirring. A methanol-acetone (1 : 2) mixture was added to precipitate (+)-bis(glycinato)(1,10-phenanthroline)cobalt(III) chloride. This was filtered and dried. Recrystallization was carried out with a methanol-acetone (1 : 2) mixture. Optical rotation was constant after two recrystallizations. Yield: 1.38 g, 62.11%; [*α*]^589^ = +138°.

#### 2.5.3. Resolution of Tris(1,10-phenanthroline)nickel(II) Chloride

A similar method as described in Section 2.5.1 was used. Potassium antimonyl-D-tartrate hydrate (3.24 g, 0.005 M) was dissolved in 10 mL of water with warming. A solution of the racemic mixture of tris(1,10-phenanthroline)nickel(III) chloride (4.54 g, 0.01 M) dissolved in 20 mL of water was then added. The diastereomer was recrystallized by dissolving in a minimum quantity of water, followed by gradual addition of methanol to produce turbidity, and then cooled in ice to obtain crystals that were filtered, washed, and dried. A solution of the diastereomer (9.21 g, 0.005 M) in a minimum amount of water was added to potassium chloride (0.51 g, 0.005 M) with vigorous stirring. A methanol-acetone (1 : 2) mixture was added to precipitate tris(1,10-phenanthroline)nickel(II) chloride as pink crystals. This was filtered and dried. Recrystallization was carried out with methanol-acetone (1 : 2) mixture. Optical rotation was constant after two recrystallizations. Yield: 1.87 g, 54.21%; [*α*]^589^ = +800°.

### 2.6. Resolution of Compounds

The geometric isomer of higher yield, which was invariably the first eluted isomer, for complexes Na[Co(L_1_)_3_], Na[Co(L_2_)_3_], Na[Cu(L_1_)_3_], and Na[Ni(L_1_)_3_], was resolved using dichlorobis(ethylenediamine)cobalt(III) chloride and bis(glycinato)(1,10-phenanthroline)cobalt(III) chloride as resolving agents using a similar procedure as described in Section 2.5.3. Attempt was also made to use tris(1,10-phenanthroline)nickel(II) chloride as a resolving agent. The general equations of reactions using (+)-dichlorobis(ethylenediamine)cobalt (III) chloride are given in

(6) NaML3++-Coen2Cl2Cl⟶+-Coen2Cl2+-ML3

(7) +-Coen2Cl2ML3+NaCl⟶+-NaML3++-Coen2Cl2Cl

### 2.7. Resolution Using (+)-*cis*-Dichlorobis(ethylenediamine)cobalt(III) Chloride

#### 2.7.1. Na[Co(L_1_)_3_] First Eluted Isomer

Sodium tris(aspartato)cobaltate(II) (2.39 g, 0.005 M) and (+)-dichlorobis(ethylenediamine)cobalt(III) chloride (1.47 g, 0.005 M). Yield: 1.88 g, 78.66%, [*α*]^589^ = +36°, ee = 28.31.

#### 2.7.2. Na[Cu(L_1_)_3_]

Sodium tris(aspartato)cupperate(II) (2.41 g, 0.005 M) and (+)-dichlorobis(ethylenediamine)cobalt(III) chloride (1.57 g, 0.005 M). Yield: 1.58 g, 65.56%, [*α*]^589^ = +47.50°, ee = 32.10.

#### 2.7.3. Na[Ni(L_1_)_3_]

Sodium tris(aspartato)nickelate(II) (2.36 g, 0.005 M) and (+)-dichlorobis(ethylenediamine)cobalt(III) chloride (1.53 g, 0.005 M). Yield: 1.53 g, 64.83%, [*α*]^589^ = +35.50°, ee = 29.60.

#### 2.7.4. Na[Co(L_2_)_3_] First Eluted Isomer (1EI)

Sodium tris(glycinato)cobaltate(II) (1.52 g, 0.005 M) and (+)-dichlorobis(ethylenediamine)cobalt(III) chloride (1.44 g, 0.005 M). Yield: 0.68 g, 42.74%, [*α*]^589^ = +42.00°.

### 2.8. Resolution Using Bis(glycinato)(1,10-phenanthroline)cobalt(III) Chloride

#### 2.8.1. Na[Co(L_1_)_3_] First Eluted Isomer

Sodium tris(aspartato)cobaltate(II) (2.37 g, 0.005 M) and (+)-bis(glycinato)(1,10-phenanthroline)cobalt(III) chloride (3.19 g, 0.005 M). Yield: 1.45 g, 61.18%, [*α*]^589^ = +34°, ee = 23.10.

#### 2.8.2. Na[Cu(L_1_)_3_]

Sodium tris(aspartato)cupperate(II) (2.38 g, 0.005 M) and (+)-bis(glycinato)(1,10-phenanthroline)cobalt(III) chloride (2.94 g, 0.005 M). Yield: 1.42 g, 59.66%, [*α*]^589^ = +47.00°, ee = 22.10.

#### 2.8.3. Na[Ni(L_1_)_3_]

Sodium tris(aspartato)nickelate(II) (2.36 g, 0.005 M) and (+)-bis(glycinato)(1,10-phenanthroline)cobalt(III) chloride (2.99 g, 0.005 M). Yield: 1.74 g, 73.73%, [*α*]^589^ = +32.00°, ee = 23.33.

#### 2.8.4. Na[Co(L_2_)_3_] First Eluted Isomer

Sodium tris(glycinato)cobaltate(II) (1.59 g, 0.005 M) and (+)-bis(glycinato)(1,10-phenanthroline)cobalt(III) chloride (3.01 g, 0.005 M). Yield: 0.49 g, 31.33%, [*α*]^589^ = +40.25°.

### 2.9. Resolution Using (+)-Tris(1,10-phenanthroline)nickel(II) Chloride

Attempt was made to resolve the complexes using (+)-tris(1,10-phenanthroline)nickel(II) chloride at complex : resolving agent ratio of 2 : 1. However, for all the complexes, the analysing lens was blurred and no rotation was observed.

### 2.10. Antimicrobial Activity Using Disc Diffusion Assay

The strains used were* Escherichia coli *NCTC 8196,* Pseudomonas aeruginosa *ATCC 19429,* Staphylococcus aureus *NCTC 6571,* Proteus vulgaris* NCIB,* Bacillus subtilis *NCIB 3610, and one methicillin-resistant* S. aureus* clinical isolate for bacteria and* C. albicans* NCYC 6 for fungi. The standard strains were from stocks of culture collections maintained at the Pharmaceutics Laboratory, Obafemi Awolowo University, Ile-Ife. The bacteria were maintained on nutrient agar slants and fungi on Sabouraud Dextrose Agar slants at 4°C and subcultured monthly. Each test agent (20 mg) was dissolved in 1 mL sterile distilled water boiled gently on a Bunsen flame. Discs of Whatman No. 1 filter paper (*φ* 6 mm) were soaked with 2 drops of the test agent using a sterile Pasteur pipette and allowed to dry at room temperature.

Two colonies of a 24-hour plate culture of each organism were transferred aseptically into 10 mL sterile distilled water in a test tube and mixed thoroughly, using an electric shaker, for uniform distribution. A sterile cotton swab was then used to spread the resulting suspension uniformly on the surface of oven-dried Mueller Hinton Agar (Oxoid) and Sabouraud Dextrose Agar plates (Sterillin) for bacteria and fungi, respectively. These were incubated for an hour at 37° and 25°C for bacteria and fungi, respectively. Sterile forceps were used to aseptically place each of the discs on the agar plates and the plates were then refrigerated for 30 min at 4°C following which the inoculated plates were incubated at 37°C for 24 hours for bacteria strains and 25°C for 72 hours for the fungal strain. Antimicrobial activity was evaluated by noting the zones of inhibition against the test organisms [50].

### 2.11. Cytotoxicity Bioassay

The procedure used was modified from the assay described by Solis et al. (1993) [51]. Brine shrimps (*Artemia salina*) were hatched using brine shrimp eggs in a conical shaped vessel (1 L), filled with sterile artificial seawater under constant aeration for 48 h. After hatching, active nauplii free from egg shells were collected from a brighter portion of the hatching chamber and used for the assay. Ten nauplii were drawn through a Pasteur pipette and placed in each vial containing 4.5 mg/L brine solution. In each experiment, different volumes of sample were added to 4.5 mL of brine solution to give different concentrations (20, 40, 60, 80, and 100 *μ*g/mL) and maintained at room temperature for 24 h under the light. The surviving larvae were counted. Experiments were conducted along with control (vehicle treated) of the test substances in a set of three tubes per dose.

### 2.12. Statistical Analysis

The in vitro antimicrobial analysis data was analysed using one-way ANOVA by SPSS 16 (Statistical Package for the Social Sciences) for Windows. Mean separation test between treatments was performed using Duncan's multiple range tests.* P *value ≤ 0.05 was considered statistically significant.

The brine shrimp mortality data was subjected to Probit Regression Analysis (Finney 1971) using the United States Environmental Protection Agency (USEPA) Probit Analysis software program version 1.5.

## 3. Results and Discussion

### 3.1. Characterization of Synthesized Complexes

#### 3.1.1. Aspartato Complexes

**Figure 3:**
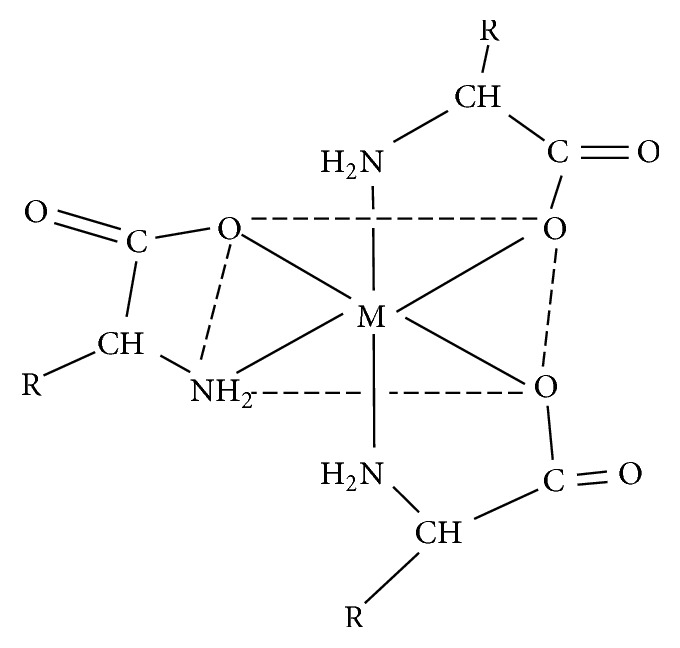
*trans*-Amine/*cis*-carboxylate isomer (R = –CH_2_COOH).

The infrared spectrum of aspartic acid showed a broad band at 3380 cm^−1^ that was assigned to the *ν*(N–H) absorption stretching frequency. On coordination, this was shifted to higher wave number with the exception of the manganese and copper complexes [52–54]. The absence of a distinct band for the manganese complex was adduced to the hydrogen bonding between the hydrogen atom of the amino substituent and the uncoordinated oxygen atom of the ligand [55, 56]. For reasons that were not quite evident, the spectrum of the copper complex exhibited no shift in the *ν*_(NH2)_ absorption when compared with that of the ligand. However, shifts in the carbon-nitrogen absorption frequency and appearance of new bands that could be ascribed to the metal-nitrogen band in the spectrum of the complex suggested that the nitrogen atom's lone pair of electrons were used for coordination in the complex (Table 1). Based on this, it was suggested that there was probable elongation of the metal-nitrogen bond, substantially for the *ν*_(NH2)_ absorption to occur at the same position. From previous studies, the axial bonds of octahedrally coordinated copper(II) complexes are of higher energy compared with the equatorial bonds; consequently, bonding at the axial position is elongated at one of the apical regions of such complex and this is attributable to the Jahn-Teller distortion [57]. It is proposed therefore that the amino substituent is positioned at the apical region in the complex; this type of arrangement may be assigned as the* trans*-amine/*cis*-carboxylate isomer of the complex (Figure 3) [52, 58]. Further buttressing this point of view is the fact that amino acids exist as zwitterions in crystalline form, with a positively charged ammonium ion with a weak *ν*(N–H) band. Similar to that obtained for the copper complex, evidence for coordination to the metal ion via the nitrogen atom of the ligand was also given by the shifts in the carbon-nitrogen absorption band frequencies on coordination for all the other complexes [52]. This was corroborated by the observed M–N absorption frequency in the region 549–599 nm, which was absent in the spectrum of the ligand [53, 59]. Intense bands at 1650 and 1583 cm^−1^ in the spectrum of the ligand were attributed to COO^−^_asy_ and COO^−^_sy_ stretching frequencies, respectively [52, 55]. On complexation, these were shifted to higher and lower wave numbers, respectively (Table 1), suggesting that the oxygen atom of the carboxylate group of the ligand was used for coordination [37, 52]. The nickel complex showed no clearly defined band and this may be attributable to the overlap of the –NH_2_ bending frequency [52]. The metal oxygen absorption frequency was observed in the region 601–682 nm (Table 1), further supporting coordination via the oxygen atom of the ligand [52–54]. Sharp extended bands in the region of 3700 and 3880 cm^−1^ suggest –OH stretching absorption with hydrogen bonding, attributed to –OH of the uncoordinated carboxylic acid of the side chain of the ligand [52, 60, 61]. This was corroborated by the presence of sharp extended bands in the 1730 cm^−1^ region in the spectrum of the complexes, attributable to free uncoordinated carbonyl stretching frequency with hydrogen bonding. As a consequence, this serves as an indication of the nonparticipation of the carboxylic acid group of the side chain in binding [56].

**Figure 4:**
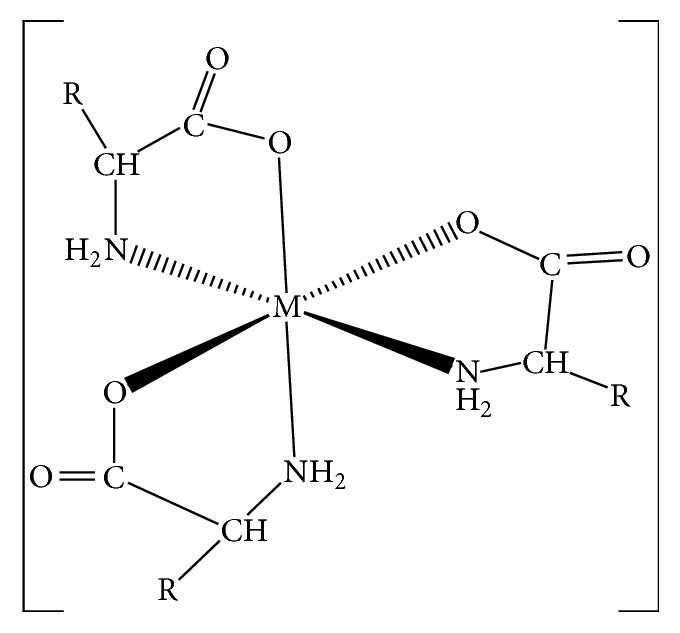
The proposed structure for the tris-chelate complexes. R = –H; glycinato complexes; –CH_2_COOH; aspartato complexes.

The electronic spectrum for aspartic acid exhibited three absorption bands at 196, 212, and 232 nm, assigned as the *n* → *σ*^*∗*^, *n* → *π*^*∗*^, and *π*^*∗*^ → *π*^*∗*^ transitions and ascribed to intraligand electronic transitions. On coordination, however, shifts were observed in these bands in addition to new* d-d* transition bands (Table 2) [33, 52, 53]. These and the magnetic moment of the complexes were used to propose probable geometry of the complexes. The electronic spectrum of the Cu(II) complex showed a well resolved band at 499 nm and a weak band at 517 nm typical for a tetragonally distorted octahedral configuration and may be assigned to ^2^B_1g_ → ^2^A_1g_ and ^2^B_1g_ → ^2^E_g_ transitions. Its magnetic moment value of 1.93 BM is indicative of a mononuclear octahedral complex. This is in agreement with what was proposed by previous workers [33, 62–66]. No* d*-*d *absorption band was observed in the spectrum of the cadmium(II) complex. A magnetic moment of zero was obtained, indicating that there was no unpaired electron. This is in accord with what was obtained by Anacona et al. (2005) [33, 67]. The spectrum for the Ni(II) complex showed* d-d *bands at 541 and 820 nm which were assigned to spin allowed transitions of ^3^A_2g_(F) → ^5^T_1g_(F) and ^3^A_2g_(F) → ^5^T_1g_(P) suggestive of an octahedral geometry [57]. The magnetic moment of 2.83 BM indicated two unpaired electrons per nickel ion, which further validates the octahedral geometry for the complex with interconversion of stereochemistries or dimerization [33]. The Co(II) complex exhibited well resolved band at 484 nm and a strong band at 505 nm, which were assigned to ^4^T_1g_(F) → ^4^T_2g_(F) and ^4^T_1g_(F) → ^4^T_1g_(P) indicative of a high-spin octahedral geometry. The magnetic moment of 5.04 BM is indicative of three unpaired electrons including orbital contribution and is in agreement with the proposed octahedral geometry (Figure 4) [33, 62, 68]. The Mn(II) complex exhibited low energy bands at 550 and 574 nm consistent with a six-coordinate octahedral geometry (Figure 4). The complex had a high-spin magnetic moment of 5.30 BM. The zero crystal field stabilization energy of the high-spin configuration confers no advantage of any particular stereochemistry for Mn(II) ion. However, the value obtained agrees well with other published work for an octahedral geometry for a d^5^ Mn(II) system [62, 63, 67].

#### 3.1.2. Glycinato Complexes

The comparison of the infrared spectra of glycine and the complexes provided evidence of coordination of the metal ion with the ligand via the nitrogen atom of its amino substituent. This is because the –NH_2_ stretching frequency for the ligand at 3119 cm^−1^ was shifted in the complexes hypsochromically to 3333 and 3428 nm for the Cu(II) and Co(II) complex, respectively. This is in accord with the concept of lone-pair donation of nitrogen atom of the ligand on coordination [52, 69]. The coordination of the nitrogen atom of NH_2_ was corroborated by the appearance of new bands, which were not present in the spectrum of the ligand, at 501 and 509 cm^−1^ ascribable to metal-nitrogen absorption frequencies [53, 59]. The observed medium band at 1112 cm^−1^ in the free ligand was attributed to the *ν*(C–N) absorption and this blue-shifted on coordination [52]. The asymmetric stretching vibration frequency observed at 1615 cm^−1^ for the carboxylate ion was shifted to higher frequencies with the complexes, confirming coordination via this functional group [52]. For the symmetric stretch, sharp extended bands were observed instead of distinct bands. This has been reported to be due to the zwitterionic nature of the ligand in the crystalline form [52]. New bands at 695 and 672 cm^−1^ were assigned to metal-oxygen absorption frequencies [33, 53, 59].

The absorption spectrum for the free ligand, glycine, exhibited bands at 199, 211, and 244 attributed to *n* → *σ*^*∗*^, *n* → *π*^*∗*^, and *π*^*∗*^ → *π*^*∗*^ transitions. The visible spectrum of the Cu(II) complex displayed bands at 620 and 632 nm assigned to ^2^B_1g_ → ^2^A_1g_ and ^2^B_1g_ → ^2^E_1g_ transitions, ascribed to a square pyramidal geometry. The magnetic moment of 1.53 BM of the glycinato Cu(II) complex is indicative of an antiferromagnetic spin-spin interaction through molecular association with possible Cu–Cu interaction or dimerization; similar results have been reported for the copper(II)acetate complex [33, 62]. Hence, these facts allowed the proposal of a dinuclear square pyramidal geometry for the complex. The Co(II) complex gave two well resolved absorption bands which were assigned to ^4^T_1g_(F) → ^4^A_2g_(F) and ^4^T_1g_(F) → ^4^T_1g_(P) transitions, respectively, consistent with a six-coordinate octahedral geometry (Figure 4). The magnetic moment of 5.20 BM of the glycinato Co(II) complex indicated a high-spin d^7^ system with three unpaired electrons which corroborates the proposed octahedral geometry (Figure 4) [33].

From previous studies, the *α*-carboxylic acid group of aspartic acid has been reported to have pK_a_ of 2.09, the *β*-carboxylic acid group pk_a_ of 3.86, and the NH_3_^+^ pK_a_ of 9.82. Hence, in aspartic acid's zwitterionic form, at pH of ~3, only the *α*-carboxylic and the amino groups are available for binding with metal ions [70, 71]. The pH range for synthesizing the complexes in this study was within the range in aspartic acid's zwitterionic form (2.31–2.44). The results obtained for the characterization of the complexes indicated that aspartic acid coordinated in a bidentate fashion, coordinating via the nitrogen of the –NH_2_ and the oxygen of the carboxylate ion. This result validates the coordination mode of aspartic acid, as a bidentate ligand [33, 37, 39]. In addition, it further corroborates the pH dependence for the available donor atoms in the ligand [37, 39, 40, 72, 73]. Glycine similarly coordinated via the same donor atoms, as a bidentate ligand.

### 3.2. Geometrical Isomers

Two geometrical isomers were obtained for Na[Co(L_1_)_3_] and Na[Co(L_2_)_3_]. From previous studies, tris-chelate complexes exhibited two possible geometric isomers, namely, the facial* cis*-*cis* and meridional* cis*-*trans* isomers. It is suggested that the more readily eluted isomer is the* cis*-*tran*s isomer. This is because it has a lower dipole moment compared to the* cis*-*cis *isomer and as such is more readily eluted from the ion-exchange column [62, 74]. The separation of the geometric isomers for [Cu(L_2_)_2_] gave two isomers as well. Dinuclear square planar complexes have been reported to exhibit* cis* and* trans* geometric isomers [75]. In this case, similar to the bis-chelate complexes, the more readily eluted isomer was ascribed to the* trans* isomer [74]. No separation was observed on the ion-exchange column for Na[Cu(L_1_)_3_] and Na[Ni(L_1_)_3_], pointing to the fact that geometric isomers are diastereomers and may be separated by physical means. Therefore, it is probable that such isomers may have been separated during preparation or purification [7, 28, 29, 39, 52, 62].

**Figure 5:**
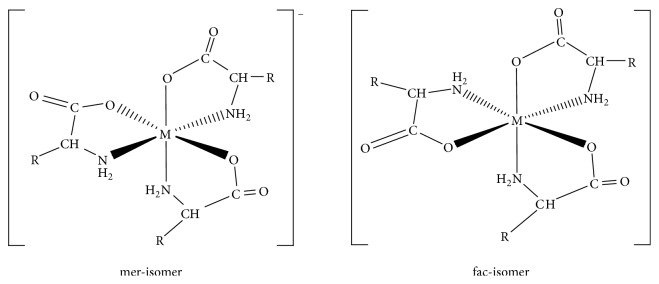
The proposed structure for the geometric isomers for the tris-chelate complexes. R = –H; glycinato complexes; –CH_2_COOH; aspartato complexes.

According to Greenwood and Earnshaw (1997), the UV-Vis spectra of geometrical isomers are often diagnostic. From this viewpoint, UV-Vis spectra of the geometric isomers for Na[Co(L_1_)_3_], [Cu(L_2_)]_2_, and Na[Co(L_2_)_3_] were obtained. The spectrum of the second eluted isomer exhibited higher molar extinction coefficient for the *d*-*d* transition absorption band than the corresponding first eluted isomer, as evident with Na[Co(L_1_)_3_] first eluted isomer (*λ*_max_ 510 nm, *ε*_510_ 40 mol^−1^ dm^-3 ^cm^−1^) and second eluted isomer (*λ*_max_ 514 nm, *ε*_514_ 250 mol^−1^ dm^-3 ^cm^−1^); with Na[Co(L_2_)_3_] first eluted isomer (*λ*_max_ 567 nm, *ε*_567_ 30 mol^−1^ dm^-3 ^cm^−1^) and second eluted isomer (*λ*_max_ 665 nm, *ε*_665_ 80 mol^−1^ dm^-3 ^cm^−1^); with [Cu(L_2_)]_2_ first eluted isomer (*λ*_max_ 628 nm, *ε*_628_ 50 mol^−1^ dm^-3 ^cm^−1^) and second eluted isomer (*λ*_max_ 637 nm, *ε*_637_ 70 mol^−1^ dm^-3 ^cm^−1^). Consequently, the second eluted isomer was assigned to the fac-isomer for Na[Co(L_1_)_3_] and Na[Co(L_2_)_3_]. This is because of the centrosymmetric nature of the mer-isomer compared to the fac-isomer (Figure 5) [52, 62, 74]. It was however assigned as the* trans* isomer for [Cu(L_2_)]_2_ and the spectrum with* d*-*d* transition bands with lower molar extinction coefficient as* cis* isomer [75].

### 3.3. Optical Activity

The specific rotation of the synthesized compounds was determined and the result obtained showed that the enantiomerically enriched compounds were dextrorotary isomers, indicating primarily that the enantiomerically enriched complexes are in excess of the dextrorotary isomer. This is not totally unexpected as the compounds were synthesized using asymmetric synthesis via chiral pool syntheses. Previous researches have shown that products obtained from such reactions are enantiomerically enriched in favour of the chiral starting material [7, 29, 38, 76]. In the case of the glycinato complexes, the specific rotation was zero, suggestive of a racemic mixture [10, 28, 29]. This may be a result of the nonchiral nature of glycine.

**Figure 6:**
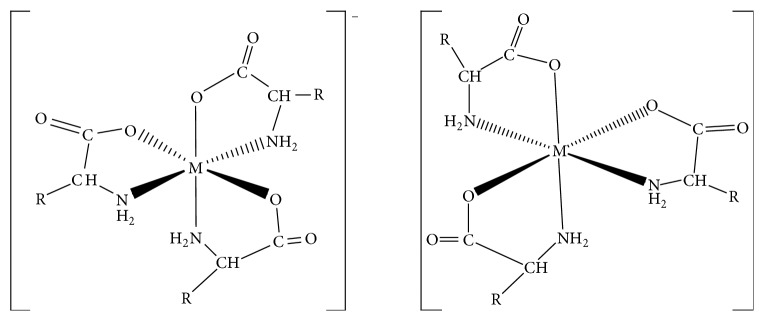
The proposed structure for the optical isomer for the tris-chelate complexes. R: –H; glycinato complexes; –CH_2_COOH; aspartato complexes.

The* mer-*isomers of Na[Co(L_1_)_3_] and Na[Co(L_2_)_3_], the synthesized complexes of Na[Ni(L_1_)_3_] and Na[Co(L_1_)_3_], were resolved using (+)-*cis*-dichlorobis(ethylenediamine)cobalt(III) complex and (+)-bis(glycinato)-1,10-phenantrolinecobalt(II) chloride as resolving agent (Figure 6). The specific rotation and enantiomeric excess of the resolved compounds were determined. The enantiomeric purity of the compounds was in the range of 22.10–23.33% using bis(glycinato)-(1,10-phenanthroline)cobalt(II) complex and 28.31–32.10% using (+)-*cis*-dichlorobis(ethylenediamine)cobalt(III) complex, the better resolving agent. Attempt to resolve the complexes using (+)-tris(1,10-phenanthroline)nickel(II) complex was not successful. According to Kirschner et al. (1979) and Lennartson (2011), tris(1,10-phenanthroline)nickel(II) complex is optically labile in aqueous solution. Therefore, it may not be an efficient resolving agent [77, 78]. From a structural point of view, we are unable to give reasons why (+)-*cis*-dichlorobis(ethylenediamine)cobalt(III) complex is the preferred resolving agent. It has however been reported that coordination compounds form diastereomeric complexes via intimate ion-pair formation [79–81]. It is proposed consequently that chiral recognition may be said to have been achieved through this mechanism. Attempt to resolve [Cu(L_2_)]_2_ using column chromatography was unsuccessful, thus suggesting the specificity of chiral resolving agents.

### 3.4. Antimicrobial Activities

The in vitro antimicrobial activities of the parent and resolved synthesized compounds are presented in Table 3. The ligands and their parent Cu(II) complexes (Na[Cu(L_1_)_3_] and Cu(L_2_)_2_) were inactive against all the tested microbes. This is contrary to the established antimicrobial activity of copper and its complexes [82–85]. Previous studies have proposed that one of the possible mechanisms of antimicrobial activity of similar compounds is via ligand exchange [31]. It is suggested that, as a result of the distortion in the geometry for (+)-Na[Cu(L_1_)_3_], it may not be well suited for the receptor site [1, 3, 31]. Hence, molecular recognition of the complex by the receptor site of the microbe may not be achieved as a consequence.

The synthesized parent complex of Na[Ni(L_1_)_3_] and the cadmium aspartato complex also lacked antibacterial activity but had similar moderate antifungal activity to acriflavine (Table 3). Also, the complexes Na[Co(L_1_)_3_], Na[Mn(L_1_)_3_], and NaCo(L_2_)_3_, respectively, had varied activities against the microbes tested. Comparing the activities of Na[Co(L_1_)_3_] and Na[Mn(L_1_)_3_] showed that they both had similar moderate activity against* P. vulgaris* and were largely inactive against* E. coli, S. aureus*, and* B. subtilis*. However, the higher activity of the latter over the former against MRSA and that of the former against* Ps. aeruginosa* and* C. albicans *may indicate the antimicrobial specificity of the complexes tested. Furthermore, the activities demonstrated by Na[Co(L_1_)_3_] against* P. vulgaris* and* C. albicans *were greatly better than those of Na[Co(L_2_)_3_] while the latter had highly better activity against* Ps. aeruginosa* and MRSA and somewhat better activities against the other two Gram-negative bacteria tested (Table 3). This may indicate that although the ligands lack antimicrobial activity, somehow, glycine contributes to the actualization of the broad spectrum antimicrobial activity of its coordinated compounds. Since glycine is more lipophilic than aspartic acid, lipophilicity is possibly a contributing factor. Lipophilicity that confers the ability to cross the membrane barrier has been reported as a factor of biological activity, including antimicrobial activity [86–95]. This suggests that chelation may serve as a tool of obtaining potent antimicrobial agents [86–93].

(+)-*cis*-Dichlorobis(ethylenediamine)cobalt(III) complex gave better enantiomeric purity and was therefore the resolving agent of choice. Compared to the parent compound, the resolved Na[Cu(L_1_)_3_] demonstrated a greatly higher activity only against* S. aureus*, a Gram-positive bacterium (Table 3). The results in Table 3 supported the hypothesis of increased biological activity of the resolved compounds and may also indicate the importance of chirality in the activity against* S. aureus* [12, 13, 40, 96]. The resolved complex of Na[Co(L_1_)_3_] gave double the zone of inhibition of the parent compound only against the Gram-positive bacteria of* S. aureus* and* B. subtilis* and a slightly better activity against the fungus and MRSA. Also, the resolved Na[Ni(L_1_)_3_] showed slightly better activities only against* E. coli* and* S. aureus* than the parent complex. Similarly, the resolved NaCo(L_2_)_3_ showed slightly better activities against* Ps. aeruginosa, P. vulgaris*, and* B. subtilis *than the parent complex (Table 3). All these results also supported the better activity of the resolved compounds and consequently the role of chirality in the demonstrated antimicrobial activity of coordinated compounds tested. On the other hand, the activities of the parent compounds of Na[Co(L_1_)_3_] against* Ps. aeruginosa* and* P. vulgaris,* Na[Ni(L_1_)_3_] against the fungus, and Na[Co(L_2_)_3_] against* S. aureus* were higher than those of the resolved compounds (Table 3). An inactive enantiomer has been suggested as a potent antagonist for a receptor site, consequently reducing the efficacy of an optically active antimicrobial agent, even at high enantiomeric excess [12, 13, 40, 96]. These results also may confirm the specificity of antimicrobial activities of the resolved compounds [12, 13, 40, 96]. Acriflavine, the standard drug, had better activities against* E. coli* and* S. aureus *than all the tested synthesized compounds while some of the latter were better against some microbes. The results obtained thus demonstrate the usefulness of resolution of racemates and enantiomerically enriched compounds in drug development of antimicrobial agents of coordinated compounds.

### 3.5. Cytotoxicity

Brine shrimp lethality bioassay is a simple cytotoxicity test based on the killing ability of test compounds on a simple zoological organism, brine shrimp (*Artemia salina*) [97]. The standard cytotoxic compound used was K_2_Cr_2_O_7_ [98]. The result obtained indicated that the complexes (parent and resolved) and the ligands elicited good cytotoxic activity with LC_50_ values in the range of 4.187 and 13.867 *μ*g mL^−1^ (Table 4). According to Meyer et al. (1982), compounds with LC_50_ < 1000 *μ*g mL^−1^ are considered significantly toxic [99]. The resolved complexes, with the exception of Na[Ni(L_1_)_3_], showed higher cytotoxicity than their respective parent complexes. Furthermore, the resolved complexes and the two ligands employed, aspartic acid and glycine, elicited cytotoxic activities in a 2–4-fold range more than the standard cytotoxic compound, K_2_Cr_2_O_7_. From the foregoing, it can be argued that the resolution process enhanced the cytotoxic properties of the synthesized complexes (Table 4). Although the tested compounds exhibited cytotoxic activity against a simple invertebrate organism, the use of appropriate human cell lines may be necessary for extrapolation of this finding to the mammalian system.

## 4. Conclusion

It was concluded from this study that the antimicrobial activity of the resolved complexes in some case was better compared to that of the parent synthesized complexes against most of the pathogens investigated. It can further be concluded that complexation imparted strong cytotoxic activities on the ligands and resolving the complexes improved on these activities. These results therefore stress the need to develop the means for complete resolution of pharmacologically active complexes in their enantiomerically enriched or racemic forms to obtain enantiopure compounds.

## Figures and Tables

**Table 1:** Relevant IR bands for the ligands and compounds.

Compound	*ν*_s_(N–H) (cm^−1^)	*ν*_asy_(COO^−^) (cm^−1^)	*ν*_sy_(COO^−^) (cm^−1^)	*ν*(M–N) (cm^−1^)	*ν*(M–O) (cm^−1^)
Aspartic acid	3380w	1650s	1583s		
Glycine	3119br	1615s			
Na[Cu(L_1_)_3_]	3380br	1666br	1547s	595br	682m
Na[Cd(L_1_)_3_]	3547,3452br	1684m	1512br	599s	658s
Na[Ni(L_1_)_3_]	3497br	—	1527w	595s	629s
Na[Co(L_1_)_3_]	3404w,br	1654s	1547s	598s	668s
Na[Mn(L_1_)_3_]	—	1681s	1506s	549s	601s
[Cu(L_2_)_2_]_2_	3333br	1632br	1416br	501s	695s
Na[Co(L_2_)_3_]	3428br	1621s	1454s	509s	672s

w: weak; m: medium; s: strong; br: broad.

**Table 2:** Electronic spectra bands for the ligands and complexes.

Compound	Band I (nm)	Band II (nm)	Band III (nm)	d-d (nm)
Aspartic acid	196	212	232	
Glycine	199	211	244	
Na[Cu(L_1_)_3_]	217	223	238, 262	499, 517
Na[Cd(L_1_)_3_]	217	238	280, 295	—
Na[Ni(L_1_)_3_]	241	310	325, 343	541, 820
Na[Co(L_1_)_3_]	223	241	247, 307	484, 505
Na[Mn(L_1_)_3_]	217	259	283, 298	550, 574, 679
[Cu(L_2_)_2_]_2_	—	265	—	620, 632
Na[Co(L_2_)_3_]	220	266	256	520, 667, 682

**Table 3:** Antimicrobial activities of the compounds.

	Zone of inhibition (size measured included 6.0 mm of the filter paper disc)											
Microorganism	Na[Cu(L_1_)_3_]		Na[Co(L_1_)_3_]		Na[Ni(L_1_)_3_]		Na[Co(L_2_)_3_]		Na[Cd(L_1_)_3_]	Na[Mn(L_1_)_3_]	[Cu(L_2_)_2_]	Acriflavine
	P	R	P	R	P	R	P	R				
*E. coli*	6.0	6.0	6.0	6.0	6.0	8.0 ± 0.9	9.0 ± 0.3	8.0 ± 0.1	6.0	6.0	6.0	20.0 ± 0.4
*Ps. aeruginosa*	6.0	6.0	11.0 ± 0.3	8.0 ± 0.2	6.0	6.0	20.2 ± 0.1	24.0 ± 0.4	6.0	8.0 ± 0.5	6.0	6.0
*P. vulgaris*	6.0	6.0	14.0 ± 0.5	6.0	6.0	6.0	9.0 ± 0.2	11.0 ± 0.6	6.0	14.0 ± 0.8	6.0	15.0 ± 0.6
*S. aureus*	6.0	16.0 ± 0.3	6.0	12.0 ± 0.2	6.0	6.0	13.0 ± 0.5	6.0	6.0	8.0 ± 0.1	6.0	20.0 ± 0.2
*B. subtilis*	6.0	6.0	9.0 ± 0.3	18.0 ± 0.8	6.0	6.0	13.1 ± 1,0	16.0 ± 0.3	6.0	8.0 ± 0.6	6.0	6.0
*MRSA*	6.0	6.0	6.0	8.0 ± 0.4	6.0	8.0 ± 0.5	15.0 ± 0.0	16.0 ± 0.2	6.0	20.0 ± 0.4	6.0	6.0
*C. albicans*	6.0	6.0	18 ± 0.2	24.0 ± 0.5	18.0 ± 0.5	9.0 ± 0.1	6.0	6.0	18.0 ± 0.5	6.0	6.0	19.0 ± 0.1

L_1_: aspartic acid; L_2_: glycine; P: parent compounds of Na[Cu(L_1_)_3_], Na[Co(L_1_)_3_], Na[Ni(L_1_)_3_], Na[Co(L_2_)_3_], and Na[Cd(L_1_)_3_]; R: parent compounds' respective resolved forms; *E. coli*:* Escherichia coli *NCTC 8196;* Ps. aeruginosa*:* Pseudomonas aeruginosa *ATCC 19429;* P. vulgaris*:* Proteus vulgaris* NCIB;* S. aureus*:* Staphylococcus aureus *NCTC 6571;* B. subtilis*:* Bacillus subtilis *NCIB 3610;* MRSA*: methicillin-resistant *S. aureus* clinical isolate;* C. albicans*:* Candida albicans* NCYC 6.

**Table 4:** Cytotoxic activity of the parent and resolved compounds.

Compound		LC_50_ (95% confidence Interval) (ug/mL)
Na[Cu(L_1_)_3_]	P	7.492 (0.003–19.817)
	R	4.691 (0.225–10.25)
Na[Co(L_1_)_3_]	P	4.576 (0.001–12.64)
	R	4.550 (0.001–12.64)
Na[Ni(L_1_)_3_]	P	4.187 (0.000–13.04)
	R	8.044 (0.477–17.49)
Na[Co(L_2_)_3_]	P	7.432 (0.882–14.92)
	R	4.775 (−)
L_1_		6.942 (1.007–13.478)
L_2_		13.867 (2.411–28.421)
K_2_Cr_2_O_7_ (Standard)		3.377 (1.479–4.505)

Values expressed as L_50_ values (95% confidence interval).

P: parent compound; R: resolved compound.
